# dilemma persist in nephrology science: developing none-melanoma skin cancer in renal transplant population

**Published:** 2013-07-01

**Authors:** Mehrdad Taghipour, Mohsen Motalebi, Behzad Einollahi, Mohammad Zaman Moradi, Arezoo Sarhangi

**Affiliations:** ^1^Nephrology and Urology Research Center, Baqiyatallah University of Medical Sciences, Tehran, Iran; ^2^Antimicrobial Resistance Research Center, Mazandaran University of Medical Sciences, Mazandaran, Iran; ^3^Medical School, Ardabil Islamic Azad University, Ardabil, Iran

**Keywords:** Kidney transplantation, Skin cancer

**Implication for health policy/practice/research/medical education:**

While transplant recipients are susceptible to develop non-melanoma skin cancer and these cutaneous lesions are the significant source of morbidity; therefore, a routine screening of these patients for skin malignancies should be performed. Phenotypic, clinical and environmental factors associated with the skin tumors following renal transplantation are different particularly due to geographic area as well as due to the study sample size, genetic variations, infectious diseases and also history of immunosuppression regimens.

An important and also vital issue that has been raised recently in Nephrology science is the risk factors for non-melanoma skin cancer in kidney transplant patients in different populations in various geographic regions.

Bernat *et al* (1), published an article that addressed this issue in it and focused its message on and drew attention to determine the association between phenotypic, clinical and environmental factors with the onset of non-melanoma skin cancer (NMSC) in kidney transplant recipients. They concluded that, in these patients, the incidence of NMSC was significantly higher than other European reports (2,3). The higher rate of skin cancer in the study of Bernat et al, may reflect several variables such as sample size, duration of follow up, type of study, geographical differences, environmental factors and immunosuppression regimens could be the causes of this apparent differences (2,3).

We agree that NMSC is considered as the most common post-transplant malignancies in Western countries (4); however, in Eastern Mediterranean area in Asia, the incidence of basal cell carcinoma (BCC) and squamous cell carcinoma (SCC) was in the next position after Kaposi’s sarcoma and non-Hodgkin lymphoma (5-7).

We performed an study on 11,255 kidney transplant recipients, in which Kaposi’s sarcoma was the most common tumor type (5). It would also disheartening to know the incidence rate of Kaposi’s sarcoma among kidney transplant recipients shows an ascending trend in the Middle East countries (6,7). In addition, we showed that the incidence of post kidney transplant malignancies was lower than Western countries and the skin cancer was occurred in 1.14% of all patients (5), which it may be partly due to geographical differences.

In the study of Bernat et al (1), 305 patients underwent kidney transplantation and 73 (25.2%) of them developed NMSC, of which the most frequent tumor were basal cell carcinoma. Most studies conducted in European countries showed that the most common skin cancer after kidney transplantation was BCC like the report of Bernat and colleagues (5-7). In contrast, studies in the Eastern Mediterranean area in Asia showed that the incidence of BCC and SCC of were in the next position after Kaposi’s sarcoma and non- Hodgkin lymphoma (8-10).

Age at time of transplant, low phototype and high pre-transplantation occupational sun exposure were showed to be as risk factors for NMSC after kidney transplantation in some studies. In our practice (5), the male sex, increased age, prolonged immunosuppression and azathioprine increased the risk of skin tumors after renal transplantation (5-10).

As we see in literature, It is possible that the incidence of malignancies and its types differs between various parts of Mediterranean region and the Middle East Asia (8). Number of multicenter studies conducted in with a large sample size in Middle East Asia countries showed that Kaposi sarcoma was the most frequent tumor in kidney transplant recipients, while these statistics are different in Mediterranean areas (9,10). In this region, the largest percentage is dedicated to NMSC such as SCC and BCC (1).

With respect to the results of recent studies (1), it should be mentioned that some factors are associated with the tumor developing after kidney transplantation such as HHV-8 and HPV infection. HHV-8 infection rate of in Mediterranean populations is intermediate (11).

In conclusion, since transplant recipients are susceptible to develop NMSC and these cutaneous lesions are the significant source of morbidity; therefore, a routine screening of these patients for skin malignancies should be performed. Phenotypic, clinical and environmental factors associated with the skin tumors following renal transplantation are different particularly due to geographic area as well as due to the study sample size, genetic variations, infectious diseases and also history of immunosuppression regimens (10-13).

## Authors’ contributions

All authors contributed to study equally.

## Ethical considerations

Ethical issues (including plagiarism, informed consent, misconduct, double publication and redundancy) have been completely observed by authors.

## Conflict of interests

The authors declared no competing interests.

## Funding/Support

No funding from any source.
